# Myopia Prevalence and Associated Factors among Secondary School Students in Finote Selam Town, Northwest Ethiopia

**DOI:** 10.4314/ejhs.v35i4.4

**Published:** 2025-07

**Authors:** Abebizuhan Zigale Bayabil, Getenet Shumet Birhan, Betelhem Temesgen Yibekal, Biruk Lelisa Eticha

**Affiliations:** 1 Department of Optometry, College of Medicine and Health Sciences, Comprehensive Specialized Hospital, University of Gondar, Gondar, Ethiopia

**Keywords:** Myopia, Secondary school, Finote Selam town, Ethiopia

## Abstract

**Background:**

Myopia is a common refractive error where light rays from distant objects focus in front of the retina when accommodation is relaxed. It is a major public health issue and one of the leading causes of blindness and visual impairment, with varying prevalence globally. This study aimed to determine myopia prevalence and associated factors among secondary school students.

**Methods:**

A school-based cross-sectional study was conducted from May 10-June 10, 2023, in which 488 participants from three secondary schools at Finote Selam town were selected by systematic random sampling. Data was collected using pre-tested, structured questionnaire and ophthalmic examination. The data were entered into Epi Data 4.6, then exported and analyzed using SPSS version 25. Descriptive analysis was summarized using frequency, percentage, and summary statistics. Binary logistic regression was used to determine the associated factors, with significance considered a p-value of less than 0.05 and a confidence interval of 95 in multivariable logistic regression.

**Results:**

A total of 488 study participants with a response rate of 98.99% were involved in the study. The mean age of subjects was 17.36 years. The prevalence of myopia was 15.37 % (95%CI: 11.93–19.10). The factors associated with myopia were gender (AOR=2.63;95%CI:1.41-4.92), residence (AOR=2.04;95%CI:1.08-3.86), family income (AOR=3.43;95%CI:1.74-6.75), family spectacle use (AOR=2.17;95%CI:l. 10-4.28), habitual working distance (AOR=2.98;95%CI: 1.17-7.58), outdoor activity (AOR=2.47;95%CI:1.22-5.02), and sleep duration (AOR=2.68;95%CI:1.28-5.59).

**Conclusion:**

considerable prevalence of myopia has been found on the study area. A total 15.37% participants had myopia. habitual working distance and high socioeconomic status were significantly associated with myopia

## Introduction

Myopia is a common type of refractive error (RE), which occurs when parallel light rays from an object at infinity are focused in front of the retina when accommodation is relaxed(1). The main clinical manifestations of myopia include reduced distance and/or near vision, impaired color vision and contrast sensitivity, narrowing of the visual field, light sensitivity, and eventually vision loss (2). Early diagnosis, treatment, and proper correction can help almost all myopia patients achieve good vision (3).

Myopia is a significant global cause of vision impairment, mainly characterized by poor uncorrected distance vision. It also reduces the quality of life related to vision and complicates activities that depend on vision(4). Additionally, myopia places a heavy burden on individuals and society, potentially affecting self-esteem, career choices, and eye health (5). Uncorrected myopia has a substantial impact on society, influencing education and the economy (6).

The cost of providing refractive correction is a significant challenge in many parts of the world, particularly in developing countries (7). The consequences of myopia for affected individuals and their families include both direct costs and lost productivity. Direct costs encompass expenses for transportation, diagnosis, treatment, and management of the condition (8). Lost productivity costs include time spent on eye exams and clinic visits, as well as unpaid caregiver time and lost work hours (8). Additionally, uncorrected refractive errors, particularly myopia, can lead to learning difficulties and reduced school performance, ultimately impacting the psychosocial development of children (9). The reasons for childhood blindness and visual impairment differ across various parts of the globe(10). Low best-corrected visual acuity has been shown to be associated with reduced ability to perform daily activities, increased risk of car accidents and falls, and increased mortality(11). School children do more close work than any other demographic, and the existence of accommodative and binocular impairment can create visual signs that disrupt work and recreational activities(12)

The number of people with myopia and high myopia is expected to increase globally. By 2050, it is projected that five billion people, which is half of the world's population, will have myopia. Out of these, around one billion people are expected to be at high risk for eye diseases that could affect their vision. In Ethiopia, studies have found myopia prevalence rates of 11.9% and 16.05%, respectively(13, 14).

There are only a few published studies on the prevalence and associated factors of myopia among secondary school students in Ethiopia. Research has looked into factors influencing myopia, such as outdoor activity, working distance, near-work activity, parental myopia, and sports (13),(15). However, there is a lack of research that explores the impact of factors like sleep duration, ocular deviation, family income, and residence on myopia. Understanding such factors can provide valuable insights for creating effective preventive and management strategies. Furthermore, no research has been conducted on the prevalence of myopia and its associated factors in the study area. Such data are essential for planning eye health care and will be valuable for policymakers in understanding and planning interventions for related issues.

The results of this study are crucial for developing early intervention plans to address the occurrence and progression of myopia among secondary school students. The broader community will also benefit from the findings this research. Health and education authorities can use the data to design regular vision screening programs and ocular health education in schools, as well as to improve eye care services within the community.

## Methods

**Study design, area and period**: This school-based cross-sectional study was conducted in Finote Selam town, located in the West Gojjam Zone, Northwest Ethiopia, between May 10 and June 8, 2023. Finote Selam is in the Amhara Regional State, 176 km from Bahir Dar City and 387 km from Addis Ababa. The town has one general hospital with a primary eye care clinic, one public health center, and five private clinics. Finote Selam comprises six kebeles (small administrative units), eight elementary schools, and three government secondary schools: Gojjam Secondary School, Damot Secondary School Number 1, and Damot Secondary School Number 2.

**Source and study population**: All secondary school students at Finote Selam Town

**Eligibility criteria**: The inclusion criteria include all secondary school students in Finote Selam town. The exclusion criteria were students with a previous history of intraocular surgery and those with an active ocular infection.

### Sample size determination

**Sample size for objective one**: The sample size was determined by using a single population proportion formula with a 95% confidence interval, and a 11.9% proportion of myopia from a similar study conducted in Gondar town, Ethiopia, was used (13). By considering the 10% nonresponse rate, the final required sample size was 493.

**Sample size for objective two**: By taking a similar study conducted in Gondar town, Ocular abnormality, and habitual working distance were identified as the primary consistent predictor for myopia and utilized to calculate the sample size(13). By using EPI INFO version 7 computer software and considering a 95% confidence level, and 80% power, the sample size was calculated as follows. A sample size of objective one was selected because it is larger and adequate to meet both objectives (Table 1).

**Table 1:** Consistent variables selected from previous study for sample size calculation of objective two

Variables	Category	Myopia		COR (95% CI)	Sample size

		Yes	No		
Ocular abnormality	No	33	394	1.00	160
	Yes	26	42	7.40 (4.03-13.52)	
Habitual working distance	>60 cm	13	153	1.00	179
	< 33 cm	32	72	5.23 (2.59-10.56)	

Note: COR: Crude Odd Ratio, CI: Confidence Interval, cm: Centimeter

**Figure 1:**
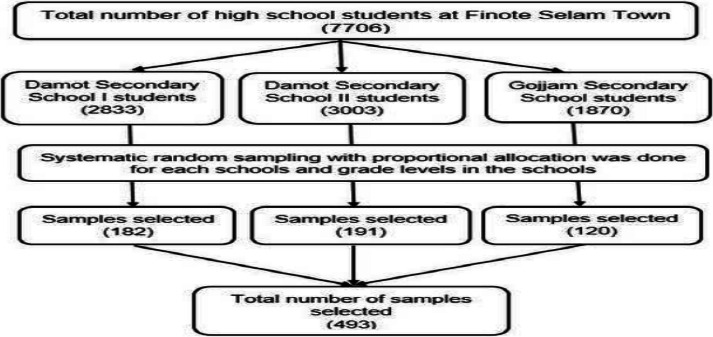
A schematic representation of the sampling technique and procedure on the prevalence and associated factors of myopia among secondary school students in Finote Selam Town, Northwest Ethiopia 2023. This diagram outlines each step of the participant selection process, including the sampling method and subsequent stages. It visually depicts the flow from identifying the target population through to the final sample unit

**Sampling technique and procedure**: There are three secondary schools in Finote Selam town, with a total of 7,706 students. All schools were included in the sampling process. The proportion of students from each school was determined using proportional allocation (i.e., the total number of students in a particular school divided by the total number of students, multiplied by the required sample size). The same proportional allocation method was used to select the number of students at each grade level within a particular school (Figure 1). The study participants were then selected through systematic random sampling with a K interval. The K interval was determined by dividing the total number of secondary school students by the total sample size (i.e., 7,706 / 493; K = 15). A random number between 1 and 15 was chosen to identify the first student, and every 15th student thereafter was included in in the sample.

### Study variables

**Dependent variable**: Myopia

**Independent variables**: This study considered various socio-demographic factors including age, gender, residence before entering secondary school, educational level, the age during the first schooling, mother and father education level, family history of spectacle use, and family economic status. Additionally, clinical factors such as ocular abnormalities, habitual working distance, and ocular alignment were considered.

Furthermore, environmental and behavioral factors including time spent on reading, daily hours of outdoor activity, outdoor sports activity, leisure activity, distance from the TV, daily hours of watching TV, daily hours of playing electronic games, sleep duration per day, row to sit in the classroom, and breaks after 30 minutes of continuous reading were taken into account.

### Operational definitions

**Myopia**: Defined as any refractive error with a spherical equivalent of at least -0.50 D in either eye (SER = sphere + 1/2 cylinder) (1).

**Outdoor activity**: Considered as “yes” if the child spends more than 2 hours per day in outdoor activities, including playing games and sports(16).

**Habitual working distance**: The habitual distance range at which a person performs near tasks, considered to be 33-60 cm. A habitual working distance shorter than 25 cm is considered a close working distance(13).

Active rest during reading: Purposely looking far into the distance for ten minutes every 40–50 minutes during study periods. This were graded as: “Occasional” (5 times every day), “Common” (6–10 times every day) and “Often” (more than 11 times every day) (14, 17).

**Ocular abnormality**: Any ocular condition (such as keratoconus, corneal ectasias, corneal degenerations and dystrophies, cataracts, lens subluxation or dislocation, and retinal abnormalities) that may cause myopia either during the condition or as a result of the disease process, as determined by an ocular examination (13).

**Secondary school students**: Students from Grade 9 to Grade 12 (13).

**Leisure activity**: Can be defined as the voluntary use of free time for activities outside the daily routine(18).

**Data collection tools and procedures**: The data collection tool was adapted from literature reviews (1),(13),(15),(19). The English version of the questionnaire was translated into Amharic and modified accordingly to facilitate understanding during the interviews. Face-to-face interviews were conducted using a pre-tested structured questionnaire, and ocular examinations were performed at the participating secondary schools during school hours. The examinations were conducted by three trained optometrists and overseen by a supervisor.

The examinations included measuring unaided visual acuity for each participant. Participants with good visual acuity (6/9 or better) underwent fogging with a +1.50 diopter (D) lens to screen for latent hyperopia. Pinhole visual acuity was measured for those with visual acuity worse than 6/12. Anterior segment examinations were performed using a pen torch, cover tests were conducted to detect any deviations, and posterior segment examinations were conducted using a direct ophthalmoscope.

Subsequently, dry static objective refraction was performed by optometrists to determine myopia status for all participants. Participants identified with ocular abnormalities or refractive errors, including myopia, were referred to Finote Selam General Hospital for further evaluation and management.

**Data processing and analysis**: The data were coded and entered into Epidata 4.6, then exported and analyzed using SPSS version 25. The investigator performed the analysis using the same software. Proportions and summary statistics, such as mean and standard deviation, were calculated for all variables.

Household socioeconomic status was measured using a wealth index constructed with principal component analysis (PCA). Variables present in less than 5% or more than 95% of cases were excluded. Factors with eigenvalues greater than 1 were extracted using PCA, and varimax rotation was applied for better interpretation. The wealth index included variables such as the source of drinking water, type of fuel, type of latrine, roof material, floor material, exterior wall material, number of rooms, household materials, livestock ownership, separate kitchen, crop production, agricultural land, and bank savings. These variables were assigned a wealth asset score and ranked into quintiles: poor, middle, and rich income. Binary logistic regression was used to determine associated factors. The Hosmer-Lemeshow test confirmed model fitness (p = 0.705). Variables with p < 0.2 in bivariable regression were included in the multivariable analysis. An adjusted odds ratio (AOR) with a 95% confidence interval (CI) was used to show the strength of association. Variables with p < 0.05 in the multivariable logistic regression were considered statistically significant.

**Data quality control**: A pre-test was conducted with 5% of the total sample size, using secondary school students from Bure Town. Necessary modifications were made based on the pre-test results. Training was provided to the data collectors. The data collection was carried out by trained optometrists, with the supervisor and the principal investigator closely monitoring the daily data collection process to ensure the completeness and consistency of the collected data in the field.

**Ethical consideration**: Ethical approval was obtained from the University of Gondar, CMHS Ethical Review Committee, in accordance with the Declaration of Helsinki. Permission to conduct the study was secured through formal letters sent to officials from the Amhara Regional Institute of Public Health, Finote Selam Town Zonal Health Office, Finote Selam Administrative Education Office, and the selected schools. Written informed consent was obtained from each student. For participants under 18 years of age, consent was obtained from their parents or guardians, and assent was requested from the children. All participants were informed about the study's purpose and their right to withdraw at any time. Confidentiality was maintained by coding the data and omitting participant names. Participants diagnosed with myopia or other ocular disorders were referred for a full ocular examination at Finote Selam General Hospital.

## Results

**Socio-demographic characteristics of the study participants**: A total of 488 students with a response rate of 98.99% participated in the study. Among study participants 298 (61.1%) were females. The mean age of the participants was 17.63 years, with a standard deviation of 1.98 years. Regarding family economic status, 133 (27.30%) study participants were from family with high economic state. Almost three-fourth 359 (73.50%) of the enrolled students came to school for the first time by their age of 7-10 years (Table 2).

**Table 2:** Socio-demographic and clinical characteristics of secondary school students enrolled under the study at Finote Selam Town, Northwest Ethiopia, 2023 (n=488)

Variables and Category	Frequency	Percent
Age of the student (in year)		
13-16	146	29.9%
17-25	342	70.1%
**Gender**		
Male	192	39.3%
Female	296	60.7%
Residence before entering secondary school		
Rural	198	40.6%
Urban	290	59.4%
Grade level		
Grade 9	158	32.4%
Grade 10	117	24.0%
Grade 11	104	21.3%
Grade 12	109	22.3%
Age during first schooling (in year)		
4-6	129	26.5%
7-10	359	73.5%
Father education		
Unable to read and write	123	25.2%
Able to read and write	152	31.1%
Primary education	58	11.9%
Secondary education	76	15.6%
College and above	79	16.2%
Mother education		
Unable to read and write	205	42.10%
Able to read and write	90	18.40%
Primary education	71	14.50%
Secondary education	64	13.10%
College and above	58	11.90%
Family history of spectacle use		
No	414	84.8%
Yes	74	15.2%
Family economic status		
Low	242	49.5%
Middle	113	23.2%
High	133	27.3%
Ocular abnormality		
No	469	96.10%
Yes	19	3.90%
Habitual working distance (in centimeter)		
<26	121	24.80%
26-30	93	19.10%
40-49	181	37.10%
≥ 50	93	19.10%
Ocular alignment		
orthophoria	470	96.40%
Esophoria	9	1.80%
Esotropia	9	1.80%

**Clinical characteristics of the study participants**: Out of 488 study participants, 19 (3.90%) students were found to have a certain type of ocular abnormality. There were 9 (1.8%) students diagnosed with esophoria and 9 (1.8%) students diagnosed with esotropia. Almost quarter of the students habitually held their reading or writing materials less than 26 cm away from their eyes (Table 2).

**Environmental and behavioral characteristics of the study subjects**: Only 71(14.50%) of the subjects experienced more than eight hours average daily reading time whereas around half of participants were not taking beak after 30 minutes of continuous reading 263 (53.90%). About half of the study subjects 198 (40.60%) and 245 (50.00%) spent less than two hours on outdoor activities and did not involve on outdoor sport activity per day, respectively. Subjects sleeping less than six hours per day constituted nearly a quarter of the total sample, totaling 115 individuals (23.60%) (Table 3).

**Table 3:** Environmental and behavioral characteristics of the study subjects at Finote Selam Town, Northwest Ethiopia, 2023 (n=488)

Variables	Frequency	Percent
Time spent on reading per day (in hour)		
< 2	42	8.6%
2-6	266	54.5%
6-8	109	22.4%
≥ 8	71	14.5%
Daily hours of outdoor activity		
< 2	198	40.6%
2-3	157	32.2%
≥ 3	133	27.3%
Outdoor sport activity		
No	245	50.2%
Yes	243	49.8%
Leisure activity		
Outdoor activity	247	50.6%
Indoor activity	241	49.4%
Distance from TV (in meter)		
No	233	47.8%
< 3	166	34.5%
3-5	66	13.5%
≥ 5	23	4.7%
Daily hours of watching Television		
No	237	48.6%
< 2	69	14.1%
2-4	160	32.8%
≥ 4	22	4.5%
Daily hours of playing electronics games		
No	170	34.8%
< 2	122	25.0%
2-4	101	20.7%
≥4	95	19.5%
Sleep duration per day (in hour)		
< 6	115	23.6%
6-7	102	20.9%
7-8	118	24.1%
≥ 8	153	31.4%
Row to sit in the classroom		
1st row	94	19.3%
2nd row	62	12.7%
3rd row	74	15.1%
4th row	63	12.9%
5th row	98	20.1%
6th row	53	10.9%
More backward	44	9.0%
Breaks after 30 minutes reading		
No	263	53.9%
< 5 times	115	23.6%
6-8 times	75	15.3%
> 8 times	35	7.2%

**Figure 2:**
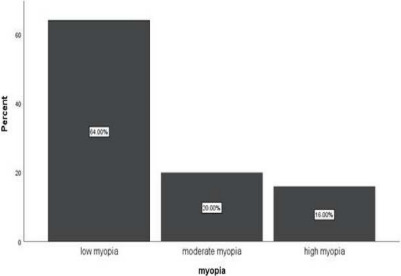
Degree of myopia of the study subjects at Finote Selam Town, Northwest Ethiopia, 2023 (n=488). This figure illustrates the various degrees of myopia identified among the participants, displaying the proportion of subjects within each severity category

Prevalence and degree of myopia: Among the students included in the study, 15.37% (95% CI: 11.93-19.10) were found to be myopic. Of these myopic secondary school students, 48 (64%) had low myopia, 15 (20%) had moderate myopia, and 12 (16%) had high myopia (Figure 2).

**Factors associated with myopia**: Once all variables were fitted to bivariable analysis separately, variables such as gender, residence before entering secondary school, age at first schooling, family history of spectacle use, family economic status, ocular abnormality, leisure activity, habitual working distance, daily hours of outdoor activity, outdoor sports activity, and sleep duration per day were confirmed to be significantly associated with myopia and entered into the multivariate logistic regression.

All of the independent variables associated with myopia in the bivariate analysis were fitted to multivariate logistic regression model. In multivariate analysis gender, residence before entering secondary school, family economic status, family history of spectacle use, habitual working distance, daily hours of outdoor activity, and sleep duration per day were significantly associated with myopia.

The odds of being myopic was 2.63 higher in females than their comparators (AOR=2.63;95%CI:1.41-4.92). Compared with being rural resident before entering secondary school, living in urban area increased the odds of myopia by above 2 fold (AOR=2.04;95%CI:1.08-3.86). Considering family economic status, being from family with high economic state increased the odds of myopia by 3.43 fold (AOR=3.43;95%CI:1.74-6.75) compared to being from economicaly poor family.

The presence of at least one person wearing spectacle for sight correction in the family made the odds of myopia occurrence on the student double than their counterparts (AOR=2.17;95%CI:1.10-4.28).

As compared to having a habit of holding reading material at distance of more than 50cm, reading habitualy at less than 26cm distance from eyes tripled the odds of being myopic (AOR=2.98;95%CI:1.17-7.58). The odds of being myopic among students spending less than two hours in a day for outdoor activity was 2.47 times higher than those stay outside for more than three hours (AOR=2.47;95%CI:1.22-5.02). Having less than six hours of average sleeping duration per day made the odds of being myopic 2.68 times higher than sleeping for more than for more than eight hours per day (AOR=2.68;95%CI:1.28-5.59) (Table 4).

**Table 4:** Factors associated with myopia in bivariate analysis and multivariate analysis among study participants at Finote Selam Town, Northwest Ethiopia, 2023 (n= 488)

Variable	Myopia		COR (95%CI)	AOR (95%CI)

	Yes	No		
Gender				
Male	21	171	1.00	1.00
Female	54	242	1.82 (1.06-3.12)	2.63 (1.41-4.92) *
Residence before entering secondary school				
Rural	18	180	1.00	1.00
Urban	57	233	2.45 (1.39-4.30)	2.04 (1.08-3.86) *
Age during first schooling				
4-6 years	25	105	1.47 (0.86-2.49)	1.11 (0.59-2.09)
7-10 years	50	308	1.00	1.00
Family economic status				
Low	32	210	1.00	1.00
Middle	10	103	0.64 (0.30-1.35)	0.90 (0.39-2.06)
High	33	100	2.17 (1.26-3.72)	3.43 (1.74-6.75) *
Family spectacle history				
No	54	360	1.00	1.00
Yes	21	53	2.64 (1.48-4.72)	2.17 (1.10-4.28)*
Ocular abnormality				
No	70	399	1.00	1.00
Yes	5	14	2.04 (0.71-5.83)	2.49 (0.79-7.83)
Habitual reading distance (in centimeter)				
< 26	27	94	3.05 (1.32-7.08)	2.98 (1.17-7.58)*
26-39	17	76	2.38 (0.97-5.82)	2.53 (0.94-6.82)
40-49	23	158	1.55 (0.66-3.61)	1.49 (0.59-3.73)
≥ 50	8	85	1.00	1.00
Daily hours of outdoor activity				
< 2	45	153	2.15 (1.16-3.99)	2.47 (1.22-5.02) *
2-3	14	143	0.72 (0.34-1.53)	0.71 (0.31-1.63)
≥ 3	16	117	1.00	1.00
Outdoor sport activity				
No	43	202	1.40 (0.85-2.31)	1.47 (0.81-2.68)
Yes	32	211	1.00	1.00
Leisure activity				
Outdoor activity	32	215	1.00	1.00
Indoor activity	43	198	1.46 (0.89-2.40)	1.25 (0.68-2.30)
Sleep duration per day (in hour)				
< 6	22	76	2.22(1.19-4.13)	2.68 (1.28-5.59) *
6-7	180	309	1.00 (0.48-2.07)	0.78 (0.34-1.81)
7-8	133	116	0.58 (0.26-1.29)	0.49 (0.20-1.20)
≥ 8	69	269	1.00	1.00

Note:

* P value <0.50

## Discussion

This study identified significant prevalence of myopia and associated factors among secondary school students at Finote Selam Town, Northwest Ethiopia.

In this study, the prevalence of myopia was 15.37% (95% CI:11.93,19.1). This study finding is comparable to studies conducted in Rural Vietnam 14.2%(6), Norway 13% (20), Ghana 18.69% (21), and Ethiopia 16.05%(14). Compared to the findings of studies conducted in Tanzania 5.6% (22), Nigeria 3.5% (23), Kenya 7.5% (24), and Ethiopia 11.9%(13), the findings of this study are higher. This discrepancy may be attributed to reduced outdoor activities and prolonged near-work activities.

However, the prevalence of myopia in this study is relatively lower compared to findings from other regions: Ghana 25.08% (25), North india 75.3% (26), South Korea 96.5% (27), and Saudi Arabia 53.5% (28). This discrepancy may be explained by the racial variation between the participants in this study and those of Asian descent. Many Asian populations have higher rates of myopia due to a complex interplay of genetic traits and environmental factors(29). Additionally, various environmental factors, particularly in developed nations with modern technology, may contribute to the occurrence and progression of myopia among students(14). An alternative explanation might be the distinct sociodemographic characteristics of the study populations.

Living in urban areas until joining secondary school increases the odds of developing myopia twofold compared to being a rural resident before starting secondary education. This finding is consistent studies conducted in India(19). The possible reason for this phenomenon might be the differences in time spent on outdoor activities versus near work activities(7). The daily lifestyle of the rural population often involves spending significant time outdoors, exposing them to sunlight. During this period, individuals are typically engaged in activities that support their families. Exposure to bright light triggers the release of dopamine from the retina, which inhibits the axial elongation of the eyeball and thereby limits the occurrence of myopia(30, 31). Additionally, living in urban areas may increase early and prolonged exposure to electronics and related near work activities(32).

Female study subjects had higher odds of developing myopia than their male counterparts. This finding aligns with reports from the Far East, including China (33, 34). This difference might be due to gender-based variations in engagement in outdoor and near work activities. Another possible reason is that females grow more rapidly than males and tend to read and write more frequently than males(35).

In the present study, students with a positive family history of spectacle use had higher odds of being myopic compared to those without such a history. Studies conducted in Ethiopia(14), rural Vietnam (6), Tanzania (22), North India (26), and Ghana (21) support our findings. This result may be attributed to a family history of myopia. However, it is impossible to conclude that a family history of spectacle use is the sole risk factor for myopia, as other refractive conditions such as hyperopia, astigmatism, and presbyopia can also necessitate spectacles. This underlines the importance of advising patients with refractive errors who wear spectacles to monitor the ocular health of their children and to bring them to eye care centers regularly.

Study participants raised in families with high economic status were three times more likely to have myopia compared to those from poor families. Studies done in India (26), China, and South Korea (36) reported same association. Typically, low socioeconomic status can place individuals at risk for poor health due to various factors, such as limited access to medical care, unsatisfactory living conditions, inadequate insurance coverage, ignorance of the negative effects of health-harming behaviors, and increased psychological stress(36). However, this may not apply to the relationship between poor health and lower socioeconomic status in the case of myopia. One reason might be that individuals seek to attain wealth through education, leading to children facing a heavier educational burden and longer years of schooling. These results in increased near-work activity and reduced outdoor activity, ultimately contributing to the occurrence of myopia(37).

Those study participants habitually holding materials within 25cm distance had higher odds of being myopic as compared to those holding at above 50cm away. The study finding is consistent with reports from India(38), rural Vietnam (6), China, and Ethiopia(13). This may be because prolonged near work leads to peripheral blurred retinal images due to ciliary spasm that occurs after extended periods of close focus. This triggers a biochemical process within the retina, sclera, and choroid, leading to axial elongation (39). A shorter reading distance can increase accommodative lag, resulting in reduced hyperopic retinal defocus and thereby promoting the development of myopia (40).

Study participants with outdoor activity of less than 2 hours per day had more likelihood of developing myopia than those spent more than three daily hours for outdoor activity. This finding is consistent with studies conducted in Norway(20), Ethiopia (16). This finding may be due to several factors. Brighter light can help reduce the development of myopia through pupil constriction, which results in a greater depth of field and less visual blur. Additionally, bright light stimulates the release of dopamine from the retina, which inhibits the axial elongation of the eyeball(41). Conversely, less outdoor activity leads to reduced light exposure and less dopamine release, which is crucial for inhibiting axial elongation(42). Consequently, this increased risk of myopia development suggests that allowing children some daily outdoor playtime could help delay the onset of myopia.

Study participants who have less than 6-hour sleep duration had high odds of being myopia as compared to those who sleep more than 8 hours per day. This finding is in agreement with the study conducted in China(34) and India(43). This findings may be due to more late-night myogenic activities, greater exposure to sub-optimal lighting, and possibly an altered circadian rhythm (44). Circadian rhythms exist in axial length, choroidal thickness, and intraocular pressure. When sleep deprivation causes disturbance to the circadian rhythm, the axial daily rhythm and the choroidal daily rhythm undergo phase shifts, leading to myopia (45). This output emplies that encouraging children to get more daily time of sleep help them protect from being myopic.

In conclusion, this research identified a 15.37% prevalence of myopia among high school students in Finote Selam Town. The study also found that several factors were significantly associated with myopia, including female gender, urban residence prior to secondary school, family history of spectacle use, higher family economic status, shorter reading distance, fewer daily hours of outdoor activity, and shorter sleep duration.

This study has some limitations. As a cross-sectional study, it does not assess changes in myopia over time or establish causal relationships between variables. Additionally, the absence of cycloplegic refraction, which is crucial for accurate measurement of refractive errors, may have led to under or overestimation of myopia prevalence due to the influence of accommodation.

## References

[1] Flitcroft DI, He M, Jonas JB (2019). IMI–Defining and classifying myopia: a proposed set of standards for clinical and epidemiologic studies. Investigative ophthalmology & visual science.

[2] Individualized M, Hard R, Vision C, Vitality A (2021). Signs and symptoms. Screen.

[3] Logan Nicola, JGaC-hT (2018). Ophthalmic & Physiologic Optics. The College of Optometrists.

[4] Flaxman SR, Bourne RRA, Resnikoff S (2017). Global causes of blindness and distance vision impairment 1990-2020: a systematic review and meta-analysis. Lancet Glob Health.

[5] Honavar SG (2019). The burden of uncorrected refractive error. Indian J Ophthalmol.

[6] al He (2020). The Prevalence of Myopia and Factors Associated with It Among Secondary School Children in Rural Vietnam. Dove Press.

[7] al Se (2019). The increasing burden of myopia in Israel among young adults over a generation: analysis of predisposing factors. Ophthalmology.

[8] Sankaridurg P, Tahhan N, Kandel H (2021). IMI impact of myopia. Investigative ophthalmology & visual science.

[9] Saxena R, Vashist P, Menon V (2013). Is myopia a public health problem in India?. Indian journal of community medicine: official publication of Indian Association of Preventive & Social Medicine.

[10] Abayo G, Gessesse GW, Asaminew T (2021). Prevalence and pattern of ocular morbidity among school children in southern Ethiopia. Ethiopian Journal of Health Sciences.

[11] Birhan GS, Belete GT, Ayele FA, Eticha BL (2024). Prevalence of Visual Impairment and Associated Factors among Adult Glaucoma Patients Attending Tertiary Eye Care Center in Gondar, Ethiopia, 2022. Ethiopian Journal of Health Sciences.

[12] Syeda SI, Kumar R, Vijayaraghavan R (2023). A comparative study to assess the accommodation and vergence relationship of myopia in Indian adolescent. Ethiopian Journal of Health Sciences.

[13] Belete GT, Anbesse DH, Tsegaye AT, Hussen MS (2016). Prevalence and associated factors of myopia among high school students in Gondar town, northwest Ethiopia, 2016. Clinical optometry.

[14] Gebru EA, Mekonnen KA (2022). Prevalence and factors associated with myopia among high school students in Hawassa City, South Ethiopia, 2019. Clinical optometry.

[15] Gebru EA, Mekonnen KA (2022). Prevalence and Factors Associated with Myopia Among High School Students in Hawassa City, South Ethiopia, 2019. Clin Optom (Auckl).

[16] Berhane MA, Demilew KZ, Assem AS (2022). Myopia: An Increasing Problem for Medical Students at the University of Gondar. Clin Ophthalmol.

[17] Wu LJ, You QS, Duan JL (2015). Prevalence and associated factors of myopia in high-school students in Beijing. PloS one.

[18] Wang H-X, Xu W, Pei J-J (2012). Leisure activities, cognition and dementia. Biochimica et Biophysica Acta (BBA)-Molecular Basis of Disease.

[19] Gopalakrishnan A, Hussaindeen JR, Sivaraman V, Swaminathan M, Wong YL, Armitage JA (2022). Prevalence of myopia among urban and suburban school children in Tamil Nadu, South India: findings from the Sankara Nethralaya Tamil Nadu Essilor Myopia (STEM) Study. Ophthalmic Physiol Opt.

[20] Hagen LA, Gjelle JVB, Arnegard S (2018). Prevalence and Possible Factors of Myopia in Norwegian Adolescents. Sci Rep.

[21] Amoah-Ayize BK, Manortey S (2022). Risk Factors of Myopia Among Students: A Case Study in Selected Garrison Junior High Schools in The Greater Accra Region. JOJ Ophthalmol.

[22] Wedner R, Todd (2002). Myopia in secondary school students in Mwanza City, Tanzania: the need for a national screening programme. Br J Ophthalmol.

[23] Akinbinu TR, Naidoo KS, Wajuihian SO (2022). Myopia prevalence in school-aged children in Garki District of Abuja, Nigeria. African Vision and Eye Health.

[24] Ragot A, Baraza M, Clarke-Farr P (2020). Prevalence of myopia and its socio-demographic distribution amongst secondary school going adolescents in Lurambi Sub-County, Kakamega, Kenya. Ophthalmology Journal.

[25] Kumah DB AN, Afoakwah P, Nelson AD, Ankamah E (2016). Prevalence of Myopia among Senior High School Students in the Kumasi Metropolis. JOJ Ophthal.

[26] Saxena R VP, Tandon R, Pandey RM, Bhardawaj A, Menon V (2015). Prevalence of myopia and its risk factors in urban school children in Delhi: the North India Myopia Study (NIM Study). PLoS One.

[27] al. Je (2012). Prevalence of Myopia and its Association with Body Stature and Educational Level in 19-Year-Old Male Conscripts in Seoul, South Korea. Invest Ophthalmol Vis Sci.

[28] al. Ae (2017). Prevalence of Myopia and its Related Risk Factors among Medical Students in Saudi Arabia. Adv Ophthalmol Vis Syst.

[29] Goss DA GT, Keller JT, Marsh-Tootle W, Norton TT, Zadnik K (1997). Optometric clinical practice guideline: care of the patient with myopia.

[30] French AN, Ashby RS, Morgan IG, Rose KA (2013). Time outdoors and the prevention of myopia. Experimental eye research.

[31] McCarthy C, Megaw P, Devadas M, Morgan I (2007). Dopaminergic agents affect the ability of brief periods of normal vision to prevent form-deprivation myopia. Experimental eye research.

[32] Yang G-Y, Huang L-H, Schmid KL (2020). Associations between screen exposure in early life and myopia amongst Chinese preschoolers. International journal of environmental research and public health.

[33] Xie Z, Long Y, Wang J, Li Q, Zhang Q (2020). Prevalence of myopia and associated risk factors among primary students in Chongqing: multilevel modeling. BMC ophthalmology.

[34] Zhao X, Lu X, Yu L (2022). Prevalence of myopia and associated risk factors among key schools in Xi'an, China. BMC ophthalmology.

[35] Grosvenor T, Grosvenor TP (2007). Primary care optometry.

[36] Lim HT, Yoon JS, Hwang S-S, Lee SY (2012). Prevalence and associated sociodemographic factors of myopia in Korean children: the 2005 third Korea National Health and Nutrition Examination Survey (KNHANES III). Japanese journal of ophthalmology.

[37] Ma Y, Lin S, Li L, Jia Y, Zou H (2021). Socioeconomic mechanisms of myopia boom in China: a nationwide cross-sectional study. BMJ open.

[38] K CG, R AVP, K K, S BK (2017). A study on prevalence of myopia and its associated factors in school children of Salem, Tamil Nadu. Int J Community Med Public Health.

[39] al. Ne (2015). Prevalence of refractive errors among junior high school students in the Ejisu Juaben municipality of Ghana. Journal of Science and Technology (Ghana).

[40] al. He (2019). The prevalence of myopia and the factors associated with it among university students in Nanjing: A cross-sectional study. Medicine.

[41] Gupta ea (2021). Outdoor activity and myopia progression in children: A follow-up study using mixed-effects model. Indian J Ophthalmol.

[42] al Re (2008). Outdoor activity reduces the prevalence of myopia in children. Ophthalmology.

[43] Binu J JR, Simon C (2016). Prevalence of Myopia and Its Associated Risk Factors among School Children in Kollam- Kerala. Int J Health Sci Res.

[44] Liu XN, Naduvilath TJ, Wang J (2020). Sleeping late is a risk factor for myopia development amongst school-aged children in China. Scientific reports.

[45] al. Xe (2023). Association between sleep-wake schedules and myopia among Chinese school-aged children and adolescents: a cross-sectional study. BMC Ophthalmology.

